# Hybridization between crops and wild relatives: the contribution of cultivated lettuce to the vigour of crop–wild hybrids under drought, salinity and nutrient deficiency conditions

**DOI:** 10.1007/s00122-012-1897-4

**Published:** 2012-06-04

**Authors:** Brigitte Uwimana, Marinus J. M. Smulders, Danny A. P. Hooftman, Yorike Hartman, Peter H. van Tienderen, Johannes Jansen, Leah K. McHale, Richard W. Michelmore, Clemens C. M. van de Wiel, Richard G. F. Visser

**Affiliations:** 1 Wageningen UR Plant Breeding, Wageningen, The Netherlands; 2 Centre for Ecology and Hydrology, Wallingford, UK; 3 IBED, Universiteit van Amsterdam, Amsterdam, The Netherlands; 4 Wageningen UR Biometris, Wageningen, The Netherlands; 5 Department of Horticulture and Crop Science, Ohio State University, Columbus, USA; 6 Genome Center, University of California Davis, Davis, USA

## Abstract

With the development of transgenic crop varieties, crop–wild hybridization has received considerable consideration with regard to the potential of transgenes to be transferred to wild species. Although many studies have shown that crops can hybridize with their wild relatives and that the resulting hybrids may show improved fitness over the wild parents, little is still known on the genetic contribution of the crop parent to the performance of the hybrids. In this study, we investigated the vigour of lettuce hybrids using 98 F_2:3_ families from a cross between cultivated lettuce and its wild relative *Lactuca serriola* under non-stress conditions and under drought, salinity and nutrient deficiency. Using single nucleotide polymorphism markers, we mapped quantitative trait loci associated with plant vigour in the F_2:3_ families and determined the allelic contribution of the two parents. Seventeen QTLs (quantitative trait loci) associated with vigour and six QTLs associated with the accumulation of ions (Na^+^, Cl^−^ and K^+^) were mapped on the nine linkage groups of lettuce. Seven of the vigour QTLs had a positive effect from the crop allele and six had a positive effect from the wild allele across treatments, and four QTLs had a positive effect from the crop allele in one treatment and from the wild allele in another treatment. Based on the allelic effect of the QTLs and their location on the genetic map, we could suggest genomic locations where transgene integration should be avoided when aiming at the mitigation of its persistence once crop–wild hybridization takes place.

**Electronic supplementary material:**

The online version of this article (doi:10.1007/s00122-012-1897-4) contains supplementary material, which is available to authorized users.

## Introduction

Gene flow between crop species and their wild relatives may result in the introgression of crop genes into wild genomic background, or in the formation of new species through novel combinations of crop and wild genes (Burke and Arnold 2001; Hails and Morley 2005). The possibility of hybridization between transgenic crops and their wild relatives has brought interest on crop–wild gene flow to another level due to the potential ecological consequences of the possibility that transgenes could also be introgressed into wild populations (Tiedje et al. 1989; Hall et al. 2000; Snow et al. 2005; Warwick et al. 2009; Wilkinson and Tepfer 2009).

Gene flow can lead to hybrid plants containing crop alleles. Crop alleles subsequently have a higher likelihood of becoming established in the population of the wild relative with few crop–wild hybridization events, when they provide a selective advantage to the fitness of the hybrid plants and their offspring than when they are selectively neutral (Lee and Natesan 2006). In the introgression and speciation processes, the unit of selection in the first generations of hybrids is not the crop gene as such, but genomic blocks from the crop consisting of the gene under selection and the surrounding linked genomic region (Stewart et al. 2003). Consequently, linkage between genes plays a crucial role in the introgression process, because a gene (or transgene) that has no effect on fitness may become introgressed just by hitchhiking along with a gene that increases fitness. Conversely, a (trans)gene could be selected against due to its proximity to a gene that reduces fitness. Such linkage would provide a natural mechanism against introgression and escape of transgenes into wild populations (Stewart et al. 2003; Kwit et al. 2011).

Multiple studies have focused on the rate of hybridization between crops and wild relatives (Arias and Rieseberg 1994; Hoc et al. 2006; D’Andrea et al. 2008; Giannino et al. 2008; Kiær et al. 2009), and on the occurrence of hybrids and their fitness in relation to the fitness of the wild parent (Snow et al. 2003; Hooftman et al. 2005, 2009; Campbell and Snow 2007). However, few studies have been conducted with the aim of understanding the specific contribution of the crop and wild parents to the fitness of the hybrids, the role of the genomic locations of the genes (as for instance assessed through quantitative trait loci (QTL), Baack et al. 2008), and the role of epistasis and genotype by environment interaction on the fitness or vigour of the hybrids. The combination of synthetic mapping populations and genetic linkage maps provides an excellent tool for studying the introgression process in an experimental setup. It allows the determination of QTLs affecting hybrid vigour or fitness, estimation of the contribution of each parent to the performance of the offspring under controlled or non-controlled conditions and monitoring of specific genomic blocks in different generations after hybridization (Rieseberg et al. 2000; Burke and Arnold 2001; Stewart et al. 2003; Baack et al. 2008).

In this study, we investigated the contribution of the crop alleles to the performance of a crop–wild hybrid population derived from a cross between cultivated lettuce (*Lactuca sativa* L.) and wild prickly lettuce (*Lactuca serriola* L.). Cultivated lettuce and wild prickly lettuce are interfertile species, the hybrids of which are viable and fertile (Lindqvist 1960; Ryder and Whitaker 1976; De Vries 1990). Experiments have shown that lettuce crop–wild hybrids are more vigorous than their parents (Hooftman et al. 2005, 2007) and that this increased vigour may lead to improved fitness of their offspring (Hooftman et al. 2009). In this study, we investigated the genetic basis of improved hybrid vigour of lettuce hybrid plants at the rosette stage. When drawing conclusions on fitness in wild populations, studies following plants during a complete cycle from seed to seed would be most optimal. However, early life stages of plants such as germination, seedling stage and vegetative growth are crucial phases as they determine the survival and reproduction of the plant, especially under stress conditions (Foolad 1996; Albacete et al. 2008; Donohue et al. 2010). In lettuce crop–wild hybrids, selection takes place on young plants, leading to surviving lineages with higher vigour and fitness than the wild genotypes (Hooftman et al. 2005, 2009). Therefore, studies of young plants could already give valuable clues on crop–wild hybrid fitness in an efficient manner through performing relatively short experiments under controlled conditions. Under natural conditions, the hybrids will most likely be subject to adverse conditions of abiotic stress such as drought, heat, cold, etc. Tolerance to abiotic stress factors is a prominent goal of today’s GM breeding and evaluation, and the release of GM crop varieties tolerant to the major abiotic stress is expected in the near future for many crop species (Castiglioni et al. 2008; Abdeen et al. 2010; Li et al. 2010; Choi et al. 2011). Therefore, we conducted experiments under controlled abiotic stress conditions of drought, salinity and nutrient deficiency in the F_2_ progeny of a cross between *L. sativa* and *L. serriola*. We addressed the following questions: (1) how is the performance of the hybrids relative to the wild parent under non-stress and stress conditions? (2) Do crop alleles contribute an advantage or disadvantage to the crop–wild hybrids under non-stress and abiotic stress conditions (drought, salinity and nutrient deficiency)? (3) How are the vigour QTLs distributed along the genome, and what is the nature of their allelic effects?

## Materials and methods

### *Lactuca serriola* and *L. sativa*

*Lactuca serriola*, prickly lettuce, is a weedy species that thrives in ruderal, anthropogenic areas (Lebeda et al. 2001). It is the closest relative of cultivated lettuce (*L. sativa*) with which it could even be considered to be conspecific (Kesseli et al. 1991; Hill et al. 1996; Koopman et al. 1998). The two species have the same number of chromosomes (2*n* = 2*x* = 18), are completely cross-compatible and the resulting hybrids are also viable and fertile (Lindqvist 1960; Ryder and Whitaker 1976; De Vries 1990). *L. serriola* and *L. sativa* therefore constitute a classic crop–weed complex perfect for introgression studies. Both species are basically autogamous, but with a limited rate of out-crossing by insects of 1 to 5 % for *L. sativa* (Thompson et al. 1958) and an interspecific hybridization rate of up to 2.5 % between the two species (D’Andrea et al. 2008). A recent large-scale population genetic study has shown the occurrence of spontaneous hybrids in the field in Europe (Uwimana et al. 2012a).

### Development of hybrid plants

F_1_ progeny was created by crossing *L. serriola* and *L. sativa* in the greenhouse. The *L. serriola* parent was a progeny of a plant collected from Eys (Province of Limburg, The Netherlands), and it represents a commonly occurring genotype among *L. serriola* in northwestern and Middle Europe (designated as “cont83” in Van de Wiel et al. 2010). For the *L. sativa* parent, we used the commercial cultivar Dynamite, a butterhead lettuce developed by Nunhems Zaden. It harbours genes for resistance to aphids, downy mildew and lettuce mosaic virus (Van der Arend et al. 1999), which represent important breeding goals of lettuce cultivars. *L. sativa* was used as the pollen donor, mimicking a scenario of pollen flow from a crop to its wild relative. Crossing was performed according to the protocols by Nagata (1992) and Ryder (1999) as described in Hooftman et al. (2005). F_2_ seeds were produced by selfing of one F_1_ plant. F_2_ seeds were sown and 200 seedlings were randomly chosen, transplanted and genotyped as described below. The plants were selfed and the resulting F_3_ seeds were harvested per individual F_2_ plant.

### Genotyping and construction of the linkage map

The Compositae Genome Project at UC Davis Genome Center has developed single nucleotide polymorphism (SNP) markers from lettuce populations derived from crosses between closely related cultivars of *L. sativa* and between *L. sativa* and *L. serriola*. These SNPs were mined initially by re-sequencing PCR-amplified genes of interest between *Lactuca sativa* cv. Salinas and *L. serriola* acc. UC96US23 using Sanger sequencing (McHale et al. 2009) and by mining Illumina sequencing data aligned to reference EST assemblies (http://compgenomics.ucdavis.edu/compositae_SNP.php). cDNA libraries from parental lines were sequenced with Illumina Genome Analyzer II. These ESTs sequences encode genes for disease resistance and plant development. In this way, more than 10,000 SNPs were developed from 3,950 ESTs in four parental pair combinations, namely Salinas × Valmaine, Pavane × Parade, Emperor × El Dorado and Thompson × Cisco (http://compgenomics.ucdavis.edu/compositae_SNP.php). To improve the conversion success rate of bio-informatically identified SNPs to molecular markers, potential SNPs were filtered to 1,083 SNPs that had been previously assayed and shown to be robust, were polymorphic in more than one of the four parental pair combinations, were not located in intron/exon splice sites, were limited to one SNP per contig, were candidate genes of interest, were evenly distributed based on previous mapping work and the ultra-dense lettuce map, and for which the surrounding sequence was suitable for oligonucleotide design for the Illumina GoldenGate assay. The selected 1,083 SNPs were converted into Custom GoldenGate Panels (OPA) for genotyping, using an Illumina BeadXpress assay. From the 1,083 SNPs, a customized OPA of 384 SNPs which were polymorphic between the F_2_ parental lines was made specifically for the population.

DNA was extracted from freeze-dried leaf samples of the 200 F_2_ and parent lines using the QIAGEN DNeasy 96 Plant Kit (QIAGEN, Venlo, The Netherlands) with slight modifications for dry plant tissue to obtain a minimum DNA concentration of 60 ng/μl. The DNA concentration was quantified using a NanoDrop 1000 Spectrophotometer V3.7 (Thermo Scientific). We genotyped 187 F_2_ individuals and the parents using the customized 384 SNP OPA in a BeadXpress assay. Out of the 384 SNPs, 355 were successfully scored in the 187 F_2_ and parental lines. 331 Markers were co-dominant, 16 were dominant for the *L. serriola* allele and 8 were dominant for the *L. sativa* allele. The genotypes for the 187 F_2_ individuals were used to build a genetic linkage map using JoinMap^®^ 4 (Van Ooijen 2006). Segregation distortion was tested against the expected allele frequency ratio of 1:1, using the χ^2^ test of goodness of fit with one degree of freedom. Markers within linkage groups were ordered using the maximum likelihood option of JoinMap (Jansen et al. 2001). The linkage map was displayed using MapChart 2.2 (Voorrips 2002).

### Greenhouse experiments

Based on the genotypes of the 187 F_2_ individuals, we selected a set of 98 F_2_ plants that optimized the number of different combinations of parental haplotype blocks, using the program “Genetic Distance Optimization” (GDOpt) (Odong et al. 2011). The program uses adapted *K*-medoids clustering (Kaufman and Rousseeuw 1990) in which one individual in each of the *K* clusters acts as cluster centre and clusters are formed by minimizing the total distance of all individuals to the nearest of the *K* individuals designated as cluster centres. In order to obtain a good starting point, the initial configuration of cluster centres was provided by a modified version of Genetic Distance Sampling (Jansen and van Hintum 2007).

F_2:3_ families were derived from the genotyped F_2_ plants by selfing, and these were used together with the parents of the cross in greenhouse experiments in Wageningen, The Netherlands. To the experimental lines, we added two additional lines, *L. serriola* acc. UC96US23 and *L. sativa* cv. Salinas, which, together with the parental lines, were later used to estimate the environmental error. We carried out two experiments: (1) the “drought experiment” (March–April 2010), which comprised drought and control treatments and (2) the “salt–nutrient experiment” (June–July 2010), which comprised salt, nutrient deficiency and control treatments. Each F_2_ plant was represented by 12 F_2:3_ seedlings per treatment. The parents and the two additional lines were also replicated 12 times per treatment.

During first establishment, the seedlings were irrigated twice a week for 2 weeks with water supplemented with nutrients. Subsequently, the treatments were started at the beginning of the third week after transplanting of the seedlings, when the plants had four to five leaves. For the drought experiment, the plants in the control treatment were still watered twice a week, while the plants in the drought treatment were not given water at all. For the salt–nutrient experiment, the plants were again irrigated twice a week, but with added 100 mM of NaCl in the irrigation water. The plants under nutrient deficiency treatment received water to which no nutrients were added. The control plants received nutrients for the whole period of the experiment. Stress was applied for 3 weeks after which time the plants were harvested at the rosette stage, 35 days after transplanting. A photoperiod of 18 °C/16 h of light and 15 °C/8 h of darkness was maintained throughout the experiments by temperature control and application of artificial lighting as needed. However, high summer temperatures influenced the greenhouse conditions during the salt–nutrient experiment when outside temperature reached as high as 35 °C.

### Phenotypic measurements

For each plant, vigour was determined by fresh and dry shoot biomass and shoot height. Vigour can be taken as a proxy for fitness at this young growth stage, but under the caveat that fitness could only be comprehensively assessed by following plants during a whole cycle from seed to seed. Shoot dry weight was measured after these were dried at 80 °C for 3 days. We also calculated shoot relative moisture content as the ratio of the amount of water in the shoot to the total shoot weight [(fresh weight − dry weight) × 100/fresh weight]. The ion content (Na^+^, K^+^, and Cl^−^) for salt and control treatments of the salt–nutrient experiment was measured. Because ion content is measured based on dry matter, the 80 °C-dried shoots were dried again at 100 °C for 24 h. The 12 plants per line per treatment were pooled, ground to fine powder, well mixed, and about 30 mg of dry matter was measured with the precise weight recorded. The ground samples were ashed at 545 °C for 5 h, diluted in 3 M formic acid and further diluted 1,000 times with extra-pure water. The diluted solutions were used in ion chromatography analysis on an 881 Compact IC pro (Metrohm AG, Herisau, Switzerland, Stolte et al. 2011).

### Analysis of phenotypic data

Statistical analysis was performed using GenStat 13 (Payne et al. 2011). Drought and salt–nutrient experiments were analysed separately. The significance of the different terms was determined by the analysis of variance, fitting the model:

$$ {\text{Response}} = {\text{general mean}} + {\text{block}} + {\text{genotype}} + {\text{treatment}} + {\text{genotype}} \times {\text{treatment}} + {\text{error}} $$

Broad sense heritability of family means of the traits was estimated for each treatment separately as the proportion of the total variance accounted for by the genetic variance using the formula

$$ H^{2} = V_{{{\text{g(F}}_{ 2} )}} /\left( {V_{{{\text{g(F}}_{ 2} )}} + V_{\text{e}} /r} \right); $$

where $V_{{{\text{g(F}}_{2} )}}$ is the genetic variance among F_2:3_ families, *V*_e_ is the environmental variance, and *r* is the number of replications (Chahal and Gosal 2002). $V_{{{\text{g(F}}_{2} )}}$ was estimated based on the restricted maximum likelihood (REML) method from the mixed model:

$$ {\text{Response}} = {\text{general mean}} + {\text{block}} + {\text{F}}_{ 2} {\text{ genotype}} + {\text{error}}; $$

with the response term representing the measured traits, and the term F_2_ genotype taken at random. *V*_e_ was the error variance derived from a one-way analysis of variance of the model:

$$ {\text{Response}} = {\text{general mean}} + {\text{block}} + {\text{parents}} + {\text{error}}; $$

with the term parents representing the two parents of the F_2_ plants and the two added lines (*L. serriola* acc. UC96US23 and *L. sativa* cv. Salinas).

### Quantitative trait loci (QTLs) analysis

In order to effectively model genotype by environment interaction (G × E, with environments represented by the different treatments) through QTL by environment interaction (QTL × E), each trait was analysed individually using the single trait—multiple environment option of the programme. Genome-wide association between markers and traits was decided based on a significance level of 0.05 corrected for multiple tests using the Li and Ji method (Li and Ji 2005). After the selection of the best variance–covariance model for the treatments (Malosetti et al. 2004), the candidate QTLs were determined by an initial genome scan. Final QTL positions were determined by composite interval mapping taking into account co-factors. The allelic effect of the detected QTLs in each treatment, the effect of QTL × E and the explained phenotypic variance of each QTL per treatment were determined by running a backward selection on the candidate QTLs in a mixed linear model, taking the QTL effect in each treatment as fixed terms and the interaction between each hybrid family and the treatment as random (Mathews et al. 2008). In that way, each QTL detected in one treatment was tested for its effect and significance in the other treatments.

Epistasis was tested for the detected QTLs (Holland 2007). Each QTL region was represented by the genotypic scores of the most significant marker in a multiple regression model in GenStat. To avoid the effect of linkage, overlapping QTLs were represented by one SNP marker and no interaction was estimated for QTLs on the same linkage group even if they did not overlap. In each treatment, every trait was explained by the main effects of all the detected QTLs to which interaction between one pair of QTLs was added at a time. QTL × QTL interaction was decided significant at a level of 0.05, which was corrected for the number of traits by the Bonferroni method (Bland and Altman 1995).

## Results

### Phenotypic variation

The analysis of variance revealed significant genotypic variation for the measured vigour traits (plant height, fresh weight, dry weight and relative moisture content; *P*_genotype_ < 0.001), and there was significant genotype × treatment variation (*P*_genotype × treatment_ < 0.001). Broad sense heritability of family means of the traits ranged from moderate to high (0.51 ≤ *H* ^2^ ≤ 0.99, Table 1), showing that the phenotypic variation among the F_2:3_ families was mainly explained by genetic factors. Heritability depended on the treatment. Plant height and relative moisture content seem to show similar heritability under controlled and stressed conditions. For the weight traits (both fresh and dry), however, heritability is consistently lower for all the stressed conditions and particularly so for drought and nutrient deficiency. Crop–wild hybridization released genetic variance: even when the means of the parents were not significantly different, heritability was relatively high as observed for dry weight under control (*H* ^2^ = 0.90) and drought conditions (*H* ^2^ = 0.66) and for relative moisture content under nutrient deficiency conditions (*H* ^2^ = 0.89) (Table 1).

**Table 1:** Mean, range values and heritability for measured traits of the F_2:3_ families and their parents under drought, salinity, nutrient deficiency and non-stress conditions

Trait	Treatment	*L. serriola* mean	*L. sativa* mean	F_2:3_ families			
				Mean	Min	Max	*H* ^2^
Plant height (cm)	Control-D	35.88	25.69	32.95	26.49	44.99	0.84
	Drought	21.63	17.85	20.52	16.80	26.43	0.82
	Control-SN	57.68	22.85	43.31	13.17	89.28	0.98
	Salt	27.08	14.72	25.68	13.34	53.53	0.99
	Nutrient deficiency	21.22	12.12	18.91	10.03	48.07	0.98
Fresh weight (g)	Control-D	44.76	72.55	53.14	31.91	69.55	0.90
	Drought	10.22	13.94	11.16	8.46	14.02	0.51
	Control-SN	34.51	55.18	42.25	28.5	53.44	0.86
	Salt	12.64	24.98	15.28	9.38	19.92	0.83
	Nutrient deficiency	7.46	10.73	8.10	5.62	10.53	0.66
Dry weight (g)	Control-D	3.08^a^	3.20	3.02	1.60	4.39	0.90
	Drought	1.83^a^	1.91	1.62	1.19	1.96	0.66
	Control-NS	2.98^a^	2.46	2.91	2.12	4.32	0.90
	Salt	1.33	1.97	1.54	1.09	2.21	0.80
	Nutrient deficiency	1.05	1.58	1.14	0.78	1.58	0.71
Relative moisture	Control-D	93.09	95.62	94.44	93.07	95.73	0.82
Content (%)	Drought	81.47	85.49	84.67	79.38	88.50	0.89
	Control-SN	91.31	95.56	93.08	88.62	94.57	0.93
	Salt	89.41	92.10	89.84	85.99	91.78	0.96
	Nutrient deficiency	85.88^a^	85.32	85.81	81.53	88.76	0.89
Na^+^ (μg/g dry weight)	Control-SN	11.02	13.24	9.19	3.18	20.70	–
	Salt	24.35	49.91	31.47	8.32	54.89	–
Cl^−^ (μg/g dry weight)	Control-SN	10.51	19.28	15.56	7.24	22.13	–
	Salt	56.37	78.47	67.78	20.65	105.67	–
K^+^ (μg/g dry weight)	Control-SN	44.14	82.77	66.14	38.36	93.07	–
	Salt	49.91	39.22	49.10	23.39	72.92	–

*Control-D* control treatment in the drought experiment; *control-SN* control treatment in the salt–nutrient deficiency experiment

^a^ *L. serriola* and *L. sativa* not significantly different

For each trait and under all the treatments, there were F_2:3_ individuals whose measurements were equal to or greater than the means of the two *L. serriola* lines (Online Resource 1). In addition, the mean for the *L. serriola* parent always fell within the range of the minimum and maximum values of the F_2:3_ families for all the traits and under all the treatments (Table 1). Therefore, among the crop–wild lettuce hybrid families, there clearly are a relevant number of examples having potentially increased vigour in comparison to the wild parent under the four tested conditions (non-stress, drought, salt and nutrient deficiency conditions).

Plant height positively correlated with biomass, except under salt treatment where fresh weight was negatively correlated with plant height (*r* = −18, Table 2). Under salt treatment, Na^+^, Cl^−^ and K^+^ negatively correlated with plant height. The correlation between ion content and plant biomass was apparently due to shoot moisture content as Na^+^ and Cl^−^ positively correlated with fresh weight and relative moisture content, but did not correlate with dry weight (*r* = 0.03 for Na^+^ and *r* = 0.07 for Cl^−^). The lack of correlation between ion content and dry weight indicates that the accumulation of ions in the shoots is not related to the biomass of the plants under salt treatment.

**Table 2:** Pearson’s coefficients of correlation among the traits

Trait	Treatment	Plant height	Fresh weight	Dry weight	Relative moisture content	Na^+^	Cl^−^
Fresh weight	Control-D	0.28					
	Drought	0.50					
	Control-SN	0.04^ns^					
	Nutrient deficiency	0.40					
	Salt	−0.18					
Dry weight	Control-D	0.35	0.83				
	Drought	0.29	0.58				
	Control-SN	0.59	0.61				
	Nutrient deficiency	0.24	0.76				
	Salt	0.33	0.77				
Relative moisture content	Control-D	−0.24	−0.19	−0.69			
	Drought	0.31	0.64	−0.17			
	Control-SN	−0.68	0.13	−0.65			
	Nutrient deficiency	0.17	0.12	−0.52			
	Salt	−0.80	0.17	−0.47			
Na^+^	Salt	−0.56	0.56	0.03^ns^	0.69		
Cl^−^	Salt	−0.77	0.65	0.07^ns^	0.80	0.79	
K^+^	Salt	−0.32	0.01^ns^	−0.13^ns^	0.23	−0.31	0.20

*Control-D* control treatment in the drought experiment; *control-NS* control treatment in the salt–nutrient deficiency experiment; *ns* correlation coefficient not significant (*P* > 0.05)

### Genotypic data

**Fig. 1:**
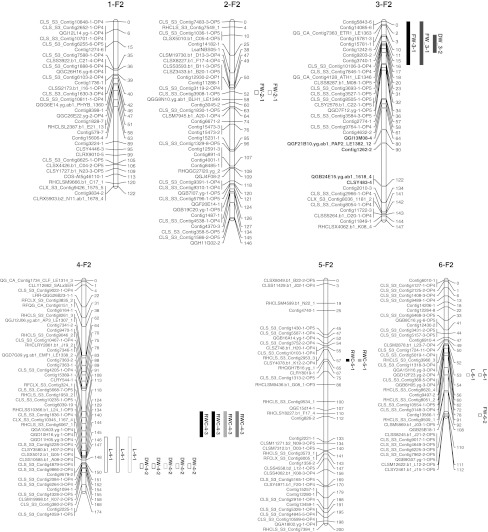

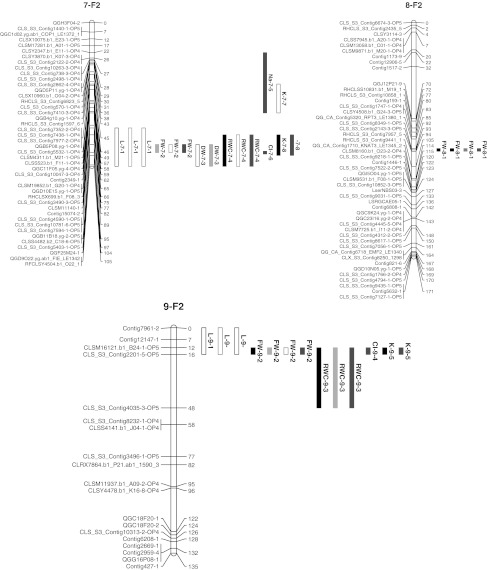
Linkage map of 345 SNPs based on 187 F_2_ plants derived from a cross between *L. sativa* and *L. serriola*. The *names* of the *markers* are shown on the *left* of the LG bar and the distance is given on the *right* in centimorgans. The *markers* with distorted segregation are shown in *red* (distortion towards the crop allele). The genomic localizations of the QTLs for plant height (L), fresh weight (FW), dry weight (DW), relative moisture content (RMC), sodium (Na), potassium (K) and chloride (Cl) as mapped under control (*black*), drought (*red*), salt (*blue*) and nutrient deficiency (*green*) conditions in 98 F_2:3 families_ are represented by the *blocks*. *Solid QTL block:* effect positive for the crop allele; *open QTL block:* effect positive for the wild allele. *Map* and QTLs displayed using MapChart 2.2 (Voorrips 2002)

The linkage map comprised 345 SNPs (Fig. 1) which, at an LOD score of 4, gave nine linkage groups (LG) representing the nine chromosomes of lettuce. These had a total length of 1,312 cM, with an individual length of 105–174 cM per LG. Each LG had 33–48 markers, with a median distance between the markers of 1.2–3.2 cM, except for LG9 that had 19 markers with a median distance between the markers of 4.2 cM.

Based on the 331 co-dominantly scored SNPs in 187 F_2_ plants, the whole crop genome was represented in the F_2_ population. The average crop allele content in the F_2_ plants was 50 % as expected, with individuals comprising 28–66 % of crop alleles. The selection of the 98 F_2_ plants for the experiment did not alter the average crop genome content. Using a significance level of 0.05 corrected for multiple tests by the Bonferroni method (α = 0.05/331, Bland and Altman 1995), eight markers (2.4 %) had crop/wild allele frequency ratios that significantly deviated from the expected 1:1 ratio (χ^2^ ranging from 14 to 65). Three of these markers could not be placed on the map and the remaining five mapped on LG3 where they spanned a continuous segment of 76 cM, with a bias towards the crop allele (Fig. 1). The flanking markers had relatively high χ^2^ values as well ($P_{{\chi^{ 2} }}$ = 0.0015) on both sides of the segment, indicating a non-random effect of segregation distortion of the segment.

### QTL analysis

Seventeen QTLs were mapped for vigour traits (plant height, fresh weight, dry weight and relative moisture content) and six QTLs were mapped for ion content traits (Na^+^, Cl^−^ and K^+^). The details about the detected QTLs under control and stress conditions are shown in Table 3 and their locations on the linkage map are presented in Fig. 1. The QTLs were located on eight linkage groups, with LG1 having no QTL. The dominance effects of the QTLs were not significant, except for two QTLs, one for fresh weight and another one for Na^+^ content, indicating that the vigour of the hybrids was not mainly due to the heterozygous genotypes. QTL by environment interaction (here, the environments represented by the treatments) was significant for all the vigour trait QTLs and Cl^−^ content QTLs. This non-additive QTL effect from one treatment to another was due to the presence of a QTL in one treatment and its absence in another or to a differential QTL allelic effect characterized by unequal or opposite allelic effect from one treatment to another.

**Table 3:** QTLs for vigour traits and ion content traits mapped under drought, salt, nutrient deficiency and non-stress conditions

Trait	QTL name^a^	Most significant marker	LG	QTL × E	Additive and dominance effect per treatment^b^ (% explained variance)				
					C-D	D	C-SN	S	N
Plant height (cm)	*L*-*4*-*1*	CLS_S3_Contig5228-3-OP4	4	Yes	−1.89 (16)	−0.54 (4)	−4.84 (8)		
	*L*-*6*-*1*	Contig13566-1	6	Yes				−3.15 (6)	−3.32 (8)
	*L*-*7*-*1*	QGB11B18.yg-2-OP5	7	Yes			−10.23 (34)	−8.66 (46)	−5.44 (23)
	*L*-*9*-*1*	CLS_S3_Contig2201-5-OP5	9	Yes			−6.06 (12)	−3.97 (10)	−5.34 (22)
Fresh weight (g)	*FW*-*2*-*1*	CLS_S3_Contig3908-1-OP5	2	Yes	*−2.84*				*−0.77*
	*FW*-*3*-*1*	Contig1242-5	3	Yes			2.52 (11)	1.09 (12)	
	*FW*-*6*-*2*	CLS_S3_Contig4649-3-OP5	6	Yes	−2.46 (5)				
	*FW*-*7*-*2*	CLSS4482.b2_C18-6-OP5	7	Yes		0.53 (12)		0.91(8)	−0.35 (6)
	*FW*-*8*-*1*	RHCLS_S3_Contig7957_5	8	Yes	3.73 (12)		2.85 (14)	0.85 (7)	0.37 (6)
	*FW*-*9*-*2*	CLS_S3_Contig2201-5-OP5	9	Yes	3.35 (9)	0.39 (6)		0.06 (11)	−0.37 (6)
Dry weight (g)	*DW*-*3*-*2*	Contig1242-5	3	Yes				0.07 (6)	
	*DW*-*4*-*2*	CLS_S3_Contig4328-2-OP5	4	Yes	−0.34 (18)	−0.07 (10)	−0.32 (20)	−0.10 (10)	−0.06 (5)
	*DW*-*7*-*3*	CLSS4482.b2_C18-6-OP5	7	Yes		0.07 (12)	−0.23 (11)		
Relative moisture content (%)	*RMC*-*4*-*3*	CLS_S3_Contig5668-7-OP5	4	Yes	0.41 (25)	1.28 (28)	0.30 (4)		0.88 (16)
	*RMC*-*5*-*1*	CLRY8019-1	5	Yes	0.24 (9)	0.40 (3)			
	*RMC*-*7*-*4*	QGB11B18.yg-2-OP5	7	Yes			0.72 (23)	0.98(32)	−0.90 (17)
	*RMC*-*9*-*3*	CLSM16121.b1_B24-1-OP5	9	Yes		0.48 (4)	0.40 (7)	0.48 (8)	
Na^+^ (μg/g dry matter)	*Na*-*7*-*5*	CLSM4311.b1_M21-1-OP5	7	NA	–	–		9.34*/3.36 (48)*	-
Cl^−^ (μg/g dry matter)	*Cl*-*7*-*6*	CLSS4482.b2_C18-6-OP5	7	Yes	–	–		10.29 (24)	–
	*Cl*-*9*-*4*	CLS_S3_Contig2201-5-OP5	9	Yes	–	–		6.71 (8)	–
K^+^ (μg/g dry matter)	*K*-*7*-*7*	CLS_S3_Contig4590-1-OP5	7	Yes	–	–		−8.44 (24)	–
	*K*-*7*-*8*	CLSS4482.b2_C18-6-OP5	7	No	–	–	5.23 (8)	5.23 (8)	–
	*K*-*9*-*5*	CLS_S3_Contig2201-5-OP5	9	No	–	–	3.68 q(5)	3.68 (5)	–

Italic values indicate significant dominance effect

*C-D* control treatment of the drought experiment, *C-SN* control treatment of the salt–nutrient experiment, *D* drought treatment, * S* salt treatment, *N* nutrient deficiency treatment, *NA* not applicable because one QTL was detected per trait

^a^QTL names are derived from the traits they determine followed by the linkage group on which they are located and the number of the QTL on that linkage group

^b^QTL effect for the crop allele; positive value: effect positive for the crop allele, negative value: effect positive for the wild allele)

Eleven QTLs were detected in the drought experiment and seven of them had a positive effect from the crop allele. Five of the QTLs were common in the control and drought treatments, while three were specific to the control treatment and three were specific to the drought treatment. Fifteen vigour QTLs were detected in the salt–nutrient experiment with five of them having a positive effect from the crop allele and three QTLs having a positive effect from the crop allele in either the control or salt treatment and a positive effect from the wild allele in the nutrient-deficiency treatment. Plant height was solely inherited from the wild parent in all the treatments, while the other vigour traits were inherited from both the crop and the wild parents.

Although the QTLs were located on eight out of nine lettuce LGs, 16 of the 23 detected QTLs were located on three LGs. These were LG4, 7 and 9 and they constituted QTL “hotspots” because the QTLs overlapped on the same segments (Fig. 1). On LG7, six QTLs overlapped on a chromosome segment of 28 cM and two more QTLs overlapped in a neighbouring region. Five QTLs overlapped on LG9 and three QTLs overlapped on LG4.

### QTL epistatic effect

Twenty-one QTL pairs epistatically affected the traits under the five treatments, increasing the explained phenotypic variance by 6–12 % (Table 4). Heterozygosity did not play an important role in the epistatic effect: for 18 QTL pairs, the predicted means for homozygous genotype combinations were equal to or greater than the predicted means for the heterozygous combinations. Four of these QTL pairs were homozygous for the crop allele, six were homozygous for the wild allele, and eight of the QTL pairs were homozygous for the crop allele at one locus and homozygous for the wild allele at the other locus.

**Table 4:** Significant QTL × QTL interactions as detected by generalized linear model analysis fitting the main QTL effects and adding interaction between one pair of QTLs at a time

Treatment^a^	Trait	QTL × QTL	% expl. variance	Predicted genotypic means^b^								
				a/a	a/h	a/b	h/a	h/h	h/b	b/a	b/h	b/b
C-D	Plant height (cm)	*L-6-1* × *RMC-5-1*	11	32.7	31.8	35.5	30.1	33.4	34.5	34.0	32.6	31.9
	Dry weight (g)	*FW-6-2* × *DW-4-2*	8	2.9	2.7	2.6	2.5	3.2	3.1	2.5	3.2	3.5
	Relative moisture content (%)	*L-4-1* × *RMC-5-1*	7	94.8	94.7	93.5	94.6	94.4	94.2	94.7	94.5	94.4
		*FW-2-1* × *RMC-5-1*	7	94.7	94.9	94.0	94.8	94.3	94.1	94.5	94.6	94.2
D	Plant height (cm)	*FW-8-1* × *DW-4-2*	12	18.2	21.3	20.4	19.7	20.8	20.2	20.7	19.4	21.8
	Dry weight (g)	*FW-3-1* × *RMC-4-3*	12	1.7	1.6	1.6	1.5	1.7	1.8	1.5	1.6	1.6
	Relative moisture content (%)	*L-6-1* × *FW-8-1*	9	85.3	84.0	84.2	83.9	85.1	86.3	83.7	84.3	85.2
C-SN	Fresh weight (g)	*L-4-1* × *L-9-1*	11	43.2	41.6	47.18	44.4	43.1	38.8	39.51	42.5	38.1
		*L-6-1* × *RMC-4-3*	11	45.7	41.0	42.9	35.8	43.1	43.5	39.2	42.1	42.0
	Dry weight (g)	*L-4-1* × *L-9-1*	9	2.9	2.8	3.2	2.8	3.1	2.8	2.4	3.0	3.1
		*L-4-1* × *L-7-1*	8	2.8	2.9	3.4	2.7	2.9	3.2	1.9	3.0	2.8
		*L-7-1* × *L-9-1*	11	2.7	2.6	2.4	2.7	3.1	3.0	2.7	3.2	3.6
		*L-7-1* × *FW-8-1*	7	2.6	2.6	3.1	3.2	2.9	2.7	3.6	3.2	2.8
	Relative moisture content (%)	*L-4-1* × *L-9-1*	6	93.4	93.2	93.1	93.6	92.83	92.8	94.0	92.9	91.9
		*L-9-1* × *DW-4-2*	6	93.2	93.6	93.7	93.3	93.0	92.7	93.3	92.9	91.6
		*DW-4-2* × *RMC-5-1*	6	94.0	93.7	92.4	93.4	93.2	93.1	92.0	92.4	92.9
N	Plant height (cm)	*L-7-1* × *L-9-1*	6	11.2	7.7	14.0	13.1	20.0	25.4	15.6	25.7	31.6
	Dry weight (g)	*L-7-1* × *L-9-1*	12	1.3	1.2	1.1	1.1	1.2	1.2	1.0	1.1	1.3
S	Plant height (cm)	*L-4-1* × *L-9-1*	6	22.9	22.4	27.0	20.7	24.9	24.9	25.5	29.2	40.8
	Fresh weight (g)	*FW-6-2* × *RMC-5-1*	7	15.4	15.5	14.6	16.2	15.5	14.4	14.5	14.7	16.6
	Relative moisture content (%)	*L-9-1* × *DW-4-2*	6	90.6	90.1	90.9	89.7	89.5	90.7	89.3	89.5	88.7

^a^ *a* homozygous for the crop allele,* b* homozygous for the wild allele, *h* heterozygous

^b^ *C-D* control treatment of the drought experiment, *D* drought, *C-SN* control treatment of the salt–nutrient experiment, *N*, nutrient, *S* salt

## Discussion

We studied the tolerance of young lettuce crop–wild hybrid plants to drought, salinity and nutrient deficiency and mapped 17 QTLs associated with plant vigour under those conditions in F_2:3_ families derived from a cross between *L. serriola* and *L. sativa*. In *Avena barbata*, early plant growth was found positively correlated to survival, fully grown plant biomass and plant fitness under field conditions (Latta and McCain 2009). In lettuce crop–wild hybrids, selection mainly takes place on young plants, leading to surviving lineages with higher vigour and fitness than the wild genotypes (Hooftman et al. 2005, 2009). Nevertheless, our results can only be taken as a first approach to assessing fitness in wild populations, as this would call for following complete growth cycles from seed to seed. We will discuss the effects of segregation distortion and QTLs affecting vigour on crop–wild introgression, and end with the possible implications for transgene dispersal mitigation.

### Segregation distortion

Interspecific crosses have been reported to result in high pre-zygotic segregation distortion in progeny (ranging from 22 to 90 % of the markers) and to be associated with reproduction barriers (Jenni and Hayes 2009; Yue et al. 2009; Platt et al. 2010). The relatively low rate of distorted segregation in the F_2_ population (2.4 %) is consistent with the close relatedness of *L. serriola* and *L. sativa* and the complete fertility between the two species (Ryder and Whitaker 1976; De Vries 1990; Kesseli et al. 1991; Koopman et al. 1998). In the same crop–wild cross, Hooftman et al. (2011) observed a segregation distortion of 7.5 % under greenhouse (no mortality) conditions. Their results are similar to ours with the differences in percentage accountable to different methods of correcting the significance level for multiple tests. The region on LG3 where the distortion was located in our study could unfortunately not be compared with the results of Hooftman et al. (2011) due to the lack of common markers. The occurrence of genomic regions at which one of the parental alleles is favoured during segregation may result in an increase in frequency of one parental allele at the expense of the other allele in subsequent generations. On one hand, further selfing of the hybrids will lead to a rapid fixation of the crop alleles in regions such as on LG3 where segregation is skewed in favour of the crop alleles, regardless of the fitness effect of the crop (trans)genes. On the other hand, regions with segregation skewed in favour of the wild alleles will slow down the crop allele fixation, although none was identified in this specific cross. The identification of such genomic regions with pre- and post-zygotic segregation distortion could be exploited to minimize the introgression likelihood of transgenes. However, those regions are relatively rare in the lettuce crop–wild crosses and the usefulness of such regions in minimizing the escape of transgenes will depend on the stability of the distortion over generations and across genotypes.

### Hybrid performance and QTL effects

Hybridization between cultivated and wild lettuce resulted in a moderate to high heritability for the vigour traits and many of the hybrids showed improved vigour over the wild parent under non-stress and stress conditions. The results suggest that, if early vigour results in better fitness, lettuce hybrids could outperform the wild parent under stress conditions of salinity, drought and nutrient deficiency. These results also are in line with previous experiments on lettuce, which have shown that crop–wild hybrids could perform equally or better than the wild parent and that, depending on their fitness, hybrids could displace the wild taxon *L. serriola* in its natural habitat (Hooftman et al. 2005, 2008). In addition, transgressive segregation was observed among the progeny of our lettuce cross, as also found in a cross of *A. barbata* ecotypes varying in drought tolerance by Latta et al. (2010). Despite the close relatedness between *L. serriola* and *L. sativa* and *L. sativa*’s most likely domestication from ancient population(s) of *L. serriola*, a recent large-scale population genetic study on crop–wild gene flow using microsatellite data on a large genebank collection and samples of wild *L. serriola* from all over Europe has shown that the two species are still for the largest part genetically distinguishable (Uwimana et al. 2012a). Improved hybrid vigour in early generations of hybrids may be associated with heterosis, which in turn could be based on dominance, overdominance and/or epistatic loci in the repulsion phase (pseudo-overdominance) (Birchler et al. 2003; Burke and Arnold 2001). Combination of epistatic and additive allelic effects from two parents at different loci in the repulsion phase has been associated with the origin of transgressive segregation that leads to the creation of superior phenotypes (Latta et al. 2010). It has been considered that this could even lead to ecologically diverging phenotypes that could invade new ecological areas, such as the sunflower hybrid species *Helianthus paradoxus* (Lexer et al. 2003a, b) and crop–wild hybrid radish (*Raphanus* spp.) (Campbell et al. 2006; Campbell and Snow 2007).

Hybrid vigour due to dominance and overdominance is expected to be short lived, as it is associated with the advantage of the heterozygote genotypes, which breaks down over subsequent generations due to selfing. In this study, additivity was the major allelic action at 16 of the 17 vigour QTLs identified in the F_2_ population. Dominance was significant for one vigour QTL (*FW*-*2*-*1*). This suggests that dominance is not the most important genetic basis behind the improved vigour among F_2:3_ families. Conversely, epistasis as a result of non-additive effect of genotypes at two QTLs was significant for the traits under stress and control conditions. Despite the proven importance of epistasis on polygenic traits (Yu et al. 1998; Tisné et al. 2010), it is often underestimated due to the required large population size, which is experimentally challenging to handle, combined with computational load, which makes it difficult to scan all pairs of loci, especially in highly heterozygous populations such as an F_2_ (Carlborg and Haley 2004). In a whole genome epistasis analysis, Bai et al. (2010) found that the interaction between identified QTLs accounted only for 18 % of all the interacting pairs of loci. We have probably also underestimated epistasis, as it was calculated only for those loci whose main effect was significant on their own and background loci were not included in the interaction analysis. Despite including only a subset of all loci in the analysis, the effect of epistasis was significant and it accounted for 6–12 % of the phenotypic variance of the traits per pair.

### QTL effects and transgene dispersal mitigation

QTLs affecting vigour negatively could be used to reduce transgene dispersal when they would be in close linkage to the transgene (Kwit et al. 2011). For a GM approach to such containment, i.e. linking the transgene for the desired trait to a gene conferring a disadvantage under natural growing conditions, such as a dwarfing gene, proofs of principle have been reported for tobacco under greenhouse conditions (Al-Ahmad et al. 2004) and for oilseed rape in the field (Rose et al. 2009). In the present study, we have been searching for genomic regions with such a gene. Many of the vigour QTLs in our stress experiments mapped to the same genomic regions, notably on LG4, LG7 and LG9. The QTL region on LG7 corresponds to the QTL for germination under low and high temperature with a positive effect from the wild allele found in the crop–wild cross Salinas × *L. serriola* UC96US23 (Argyris et al. 2005). It also overlaps with the QTL for the number of lateral roots in the bottom length of the taproot with a positive effect from the wild allele in the same cross (Johnson et al. 2000). Co-localizing QTLs were also obtained by Baack et al. (2008) for traits related to survival and morphology in a recombinant inbred line population of crop–wild sunflower hybrids. QTL co-localization may be due to a pleiotropic effect, if one QTL affects more than one trait, but it is also possible that the QTLs are genetically linked and inseparable with the markers and recombination events observed in this study. The combination of QTL hotspots with QTL × treatment interaction through opposite allelic effect across treatments makes it difficult to choose which QTL region favours which parental allele. Nevertheless, these regions will remain under selection, positively or negatively, depending on to the prevailing conditions (optimum, dry, saline or nutrient deficient). Therefore, as insertion site of a transgene, such QTL regions could better be avoided because there is always a chance that the regions happen to come under positive selection, leading to an increased frequency of linked loci through genetic hitchhiking, and thus to a higher likelihood of introgression of crop alleles or transgenes into the wild population (Stewart et al. 2003; Hooftman et al. 2011; Kwit et al. 2011). As a consequence of the “hotspots” of vigour QTLs, examples of QTLs with apparently more simple implications for transgene presence were relatively few, e.g. on LG2 where only wild alleles were favoured and LG8 where only crop alleles were favoured. The LG8 QTL could thus clearly be better avoided, whereas the LG2 QTL could be hypothesized to be a safer place, but as there were only two conditions with the wild allele effect, this has only weak support (see Fig. 1). From F_1_ progeny, the natural process of introgression in lettuce will continue with the creation of inbred lines through continued selfing or backcrosses to *L. serriola*, or a combination of the two. In an accompanying study, QTLs were also assessed in BC_1_ and BC_2_, where similar QTL “hotspots” were found as in the F_2_ (Uwimana et al. 2012b). With regard to the above examples, the LG8 QTL with positive crop allele effects were confirmed, but the LG2 QTL was not found.

This study was limited to a single cross and to measuring plant vigour at an early stage of growth of the hybrid plants under controlled greenhouse conditions, while spontaneous crop–wild hybrids grow under natural field conditions. Additionally, greenhouse and field experiments are not always consistent (Gardner and Latta 2008; Latta and McCain 2009). Hence, in follow-up experiments hybrids from another cross are evaluated as well, and the hybrids are evaluated on the field in order to correlate early vigour with adulthood and reproduction, and link individual stress treatment with field conditions, which may encompass multiple abiotic stress factors in combination with biotic stress factors such as diseases and herbivores.

## Electronic supplementary material

Below is the link to the electronic supplementary material.

Supplementary material 1 (DOC 91 kb)
