# Supplementary material for: Hybridization between crops and wild relatives: the contribution of cultivated lettuce to the vigour of crop–wild hybrids under drought, salinity and nutrient deficiency conditions
Source: Theor Appl Genet. 2012 Jun 4;125(6):1097–111. doi: 10.1007/s00122-012-1897-4 (PMC3442173; doi:10.1007/s00122-012-1897-4)
Supplement: Supplementary file 1 — Supplementary material 1 (DOC 91 kb) [file 122_2012_1897_MOESM1_ESM.doc]

Online Resource

1Boxplots showing phenotypic variation among F_2:3_ plants (position 1), *L. serriola* acc. UC96US23 (position 2), *L. sativa* cv. Salinas (position 3), *L. serriola/*Eys (position 4) and *L. sativa* cv. Dynamite (position 5) for dry weight (A), fresh weight (B), plant height (C) and relative moisture content (D) under the five treatments: for all the vigour traits and under stress and non-stress conditions, there are hybrid plants that perform equally as or better than the two L. serriola genotypes

**Drought**

**Control_Drought**

**Nutrient deficiency**

**Control_Salt-Nutrient**

**Salt**

**D**

**A**

**B**

**C**
